# Society Proceedings

**Published:** 1902-02

**Authors:** 


### SOCIETY PROCEEDINGS.

### ANNUAL MEETING OF THE VETERINARY MEDICAL ASSOCIATION OF NEW JERSEY.

The eighteenth annual meeting of the Veterinary Medical Association of New Jersey was held at the Trenton House, Trenton, N. J., on January 9, 1902.

The meeting was called to order at 10 A.M., by the President, Dr. William Herbert Lowe, of Paterson.

Members present: The following members were in attendance: Drs. A. Brown, of Windsor; T. Earle Budd, of Orange; George E. Fetter, of Hopewell; James T. Glennon, of Newark; G. P. Harker, of Trenton; E. A. Hogan, of Newark; J. B. Hopper, of Ridgewood; L. P. Hurley, of Hopewell; E. L. Loblein, of New Brunswick; S. Lockwood, of Woodridge; William Herbert Lowe, of Paterson; J. Payne Lowe, of Passaic; J. V. Laddey, of Arlington; James T. McDonough, of Montclair; James M. Mecray, of Maple Shade; George W. Pope, of Athenia; Werner Runge, of Newark; T. E. Smith, of Jersey City; A. T. Sellers, of Camden; S. S. Treadwell, of Englewood, and L. E. Tuttle, of Bernardsville.

Visitors and delegates present: Dr. Robert W. Ellis, of New York City, President of the Veterinary Medical Association of New York County; Drs. Leonard Pearson and W. Horace Hoskins, of the Pennsylvania Association; J. M. W. Kitchen, M.D., of East Orange and Dr. T. B. Rogers, of Woodbury.

President Lowe gave his annual address, which was pithy with good suggestions to committee-men and members in general.

Applications for membership were received from Drs. Wm. P. Fink, of Atlantic City; Jacob Bühler, of West Hoboken, and Edward Rowe, Jr., Health Officer for the city of Summit.

Applicants were duly vouched for and approved by the Board of Censors and were unanimously elected to membership.

Dr. Werner Runge, chairman, presented the report of the Public Health Committee, which is here given in part:

"Dr. John V. Laddey has prepared a paper, entitled, 'The X-Ray as an Aid in the Diagnosis of Tuberculosis in Cattle.' Dr. Werner Runge will submit a paper entitled 'Results of my Experiments to Transmit Tuberculosis of Man to the Bovine Race.' The work of the Public Health Committee is of a broad character and can only be covered in part. The committee recommends the following resolutions:

"*Resolved*, That the Members of Congress and State Legislatures be requested to provide suitable laws establishing a commission to be composed of the best men available, empowering said commission to make use of condemned criminals as subject for experimenting in the transmission of bovine tuberculosis to mankind.

"*Resolved*, That no cattle should be allowed to drink from any polluted pond, brook, or river. All public water troughs should be so constructed as to allow constant circulation of the water contained therein; that the overflow of said trough should be so constructed as to allow all water to flow from the sides of the same, thereby carrying off the surface water of the said troughs continually.

"*Resolved*, That we, as members of the Association, do hereby declare ourselves as heartily in favor of carrying out the law making it compulsory on the part of all veterinarians to report glanders whenever it comes under their observation. And we further suggest that the law be carried out stringently, and that the party concealing any case of glanders coming under his observation be summarily dealt with.

"*Resolved*, That it is the sense of this Association that every city, town, or township Board of Health should have at least one veterinarian on its staff, whose duty it shall be to have charge of contagious diseases of animals and the inspection of meat and milk.

"WERNER RUNGE,
"J. V. LADDEY,
"G. F. HARKER."

In the discussion following the reading of the report special interest centred upon the recommendation made regarding experimental work upon criminals.

Dr. Pearson spoke of the personal responsibility of physicians who experiment upon human beings, and asked if a physician who by injecting bovine tubercle bacilli into a human body and thus causing disease and death be adjudged guilty, what can be said of the editors of agricultural papers who have been recommending owners to keep tuberculous cows and use or sell the products from such animals? He believed that public opinion was against experimenting on human beings, that it would prejudice the public against medical men and shake public confidence in them, and would be morally wrong. Experiments should be limited to animals.

Dr. Laddey, speaking for the recommendation of the committee, argued that in cases of experimenting as suggested by the committee, the law would place the responsibility upon the State, and a physician would be acting for the State, and hence not personally responsible for the result of any experiment. Dr. Treadwell argued that such a law could not prevail in this country, where all men are born free and equal.

Dr. Rogers stated that there was danger of a misconception of the fundamental principles of law; that we should remember the objects for which criminals were punished, among them being the protection of society and the reformation of the criminal.

Dr. Kitchen argued that the air which we breathe is laden with germs and that they are not to be so greatly feared as some might believe; that autopsies reveal the fact that tuberculosis exists or has existed at some period of life in a large percentage of the human family, and that it might not be such a serious matter for a person in good health and with great resistive power to subject himself to experiments, and that the State might better pay volunteers than experiment with criminals.

Dr. Hoskins gave warning that such a measure as had been suggested would meet with much adverse criticism in this country, where many persons were even opposed to experimental work upon animals; that such a radical movement in the profession would be freighted with great danger.

At 1 P.M. the discussion was closed and adjournment taken for dinner.

Upon reconvening the report of the Animal Industry Committee was called for and given by the chairman, Dr. J. Payne Lowe, and was in substance as follows:

"Recommended that competent veterinarians be officially connected with all agricultural and live-stock fairs, horse shows, and dog shows, and that an animal census of the State be taken.

"Suggested that the subject of transportation of animals be looked into; the cars used for transporting animals, their construction and equipment, light and ventilation, cleanliness, disinfection, manner of feeding, watering, and caring for stock are some of the details that the Association should investigate. There are a large number of horses and cows shipped into this State, cows advanced in pregnancy and lost either at the time of parturition or afterward, due to the effect of a retained placenta, metritis, etc., where the cause can be traced directly to rough treatment received and exposure during transportation. Your committee would recommend that all stallions and bulls be periodically examined as to soundness and health by a competent veterinarian appointed by the proper State or county authorities, and that before said stallions and bulls be allowed to stand for breeding purposes the owners of such animals be required to produce a certificate of such veterinary examination. There are a number of diseases (such, for example, as osteoporosis in horses) that, from an economic and agricultural stand-point, concern the people of the State, and which the veterinary profession must deal with, and your committee is of the opinion that the State—State Agricultural College and Laboratory—should make original investigations as to the etiology and pathology of this class of diseases. We would further recommend that this Association urge upon the proper authorities the necessity of asphalt and similar pavements being sprinkled with sand during the slippery season, as many animals are severely injured by falls, etc. This committee would like, at a later date, to take up the subject of foods and feeding, with relation to the application of scientific principles in feeding animals to produce the best and most economic results."

Signed for the committee,

J. PAYNE LOWE,
Chairman.

Dr. Budd, chairman of the Committee on Legislation, reported for that committee, and recommended that the Association indorse a bill which had been drafted by the committee, the said bill providing for the creation of a State Board of Veterinary Medical Examiners to regulate the practice of Veterinary Medicine, Surgery, and Dentistry in the State of New Jersey. The bill was read and freely discussed by members, and it was finally voted that the committee be authorized to draw upon the treasury for the needed funds and assume full charge of introducing the bill in the Legislature.

Dr. Sellers, chairman of the Committee of Arrangements for the Atlantic City meeting, reported for the committee.

The report was accepted and the committee discharged, with thanks.

Dr. J. V. Laddey, delegate to the State Sanitary Association, reported, and his report was received and filed.

Dr. William Herbert Lowe reported as a delegate to the meeting of the New York State Society. It was moved and carried that a vote of thanks be extended to the veterinarians of Pennsylvania and New York for the assistance rendered this Association in entertaining the National Association at the Atlantic City meeting.

Drs. Treadwell, Sellers, and Budd, appointed a committee on resolutions, presented the following, which were unanimously adopted by a rising vote:

"WHEREAS, God in his wise providence has taken away our brother and fellow-laborer, Rush S. Huidekoper, who had for many years been a faithful worker in our profession, be it

"*Resolved*, That we hereby express our appreciation of his high personal character, his wise counsel, his long-continued interest in the veterinary profession, and his ever willing and substantial help. With deepest sympathy for his bereaved family, we commend them to God, the only comfort in such an hour of trial; be it further

"*Resolved*, That a copy of these resolutions be sent by the Secretary to the widow and family of our deceased brother."

J. M. W. Kitchen, M.D., of East Orange, N. J., who is interested in the dairy industry, read a paper entitled "Some of the Unsolved Problems of Milk Fever." The paper was well received and quite generally discussed; the conclusions reached were that while the disease was probably caused by ptomaine poisoning originating in the udder, no one had as yet succeeded in demonstrating the fact positively, and that laboratory experiments should be conducted by the State and Government, with a view of determining the exact nature of the disease, the expense of such experimenting being beyond the reach of the average owner of live-stock.

A paper entitled "The X-Ray as an Aid in the Diagnosis of Tuberculosis" was presented by Dr. J. V. Laddey, who has been making some practical use of an X-ray machine in the work of inspecting cattle for slaughter. Dr. Laddey illustrated his paper by means of ante-mortem and post-mortem photographs of animals subjected to the experiment.

The paper appears on page 97.

The Public Health Committee was requested to secure, if possible, an X-ray apparatus for use at the next meeting.

Dr. Werner Runge reported some personal experiments conducted by him during the past few months, and his report, which is of great interest, is here given in full:

"*To the President and Members of the Veterinary Medical Association of New Jersey:*

"GENTLEMEN: The statement made by Prof. Koch, M.D., at the British Congress on Tuberculosis, held in London, England, June 23, 1901, that human tuberculosis cannot be transmitted to the bovine race, is an error and without any practical foundation. It has not only aroused the profession in general, but medical and laymen have become more or less interested in the matter. In view of these facts the members of our profession should use their utmost efforts in investigating the above statement.

"Through the kindness of Mr. Stephen Francisco, of the Fairfield Dairy, Caldwell, N. J., cattle have been placed at my disposal, as well as the use of the Isolation Hospital at the said dairy, for experimental purposes. Also, through the valuable aid of Dr. R. N. Connolly, Bacteriologist for the Newark Board of Health, I was assisted in selecting a specimen of virulent sputum of a young person affected with acute tuberculosis.

"On August 14th last I selected a yellow cow that gave no reaction to retest, nor had it done so to a previous tuberculin test made November 12, 1900, and injected the same hypodermically on August 29, 1901, with the above-mentioned sputum. At both shoulders and both flanks I made also four injections into a calf five weeks old, born in a pasture lot and kept in the said lot until the day of injection, the mother of which, previous to the birth of the said calf, giving no reaction to the tuberculin test. As all cattle of this dairy, about 600 in number, are tested previous to their coming into the herd, and are rejected if they give any reaction at all, it is reasonable to believe that this calf was free from tuberculosis, as it had never been exposed to the same. The temperature of both animals, taken twice daily after the injection of the sputum, did not show anything abnormal, and no symptoms of any disease were revealed except some enlargement of the retropharyngeal glands. The calf was killed November 14, 1901, eleven weeks after injection. The cow was killed November 20, 1901, about twelve weeks after injection. The post-mortem showed the following lesions:

"The calf killed November 14th did not show any marks at the place of injection, but enlarged tubercular bronchial glands, several of which were broken down and calcareous. A large number of gray nodules were visible in the lung tissue, also one cyst, about the size of a small hen's egg, and full of pus. The microscopical examination of the scraping of the walls of the cyst, as well as the scraping of the interior wall of the broken-down gland, showed the bacilli of tuberculosis in large numbers.

"The cow killed November 20th had a large cyst full of pus at the right shoulder, and an induration of the tissue on the left side at the places of injection. The bronchial glands were enlarged and partly broken down and full of calcareous substance. The microscopical examination of the scraping of the interior of the wall of the said broken-down glands also showed tubercle bacilli. Respectfully submitted,

"WERNER RUNGE."

The members expressed their appreciation of Dr. Runge's efforts in experimental work, and among those who joined in the discussion were Drs. Pearson, Hoskins, Kitchen, and Rogers.

Dr. James T. McDonough, of Montclair, here read a paper entitled "The Horse's Foot." Dr. McDonough's paper was well written and upon a subject with which he is especially conversant.

As the hour was late it was voted that a discussion of the paper be deferred until the next meeting.

At 5.30 the meeting adjourned. The next meeting will be held at Newark, N. J., in July. GEORGE W. POPE,
Secretary.

### KEYSTONE VETERINARY MEDICAL ASSOCIATION.

THE January meeting was held at the northwest corner of Broad and Filbert Streets, Philadelphia, Pa., January 14, 1902, with the following members of the profession in attendance: Drs. Cox, Harger, Williams, Carter, Underhill, Lintz, Marshall, Noack, and Ranck, also about twenty students of the Veterinary Department, University of Pennsylvania. After the regular business was transacted we listened to valuable papers by Dr. C. J. Marshall and Dr. S. J. J. Harger.

The object of Dr. Marshall's paper in part was to prevent our brothers in the veterinary profession from making flippant remarks, either to other members of the profession or to our clients, about the uselessness of certain drugs, instruments, or operations, especially those which have stood the tests for hundreds of years and proved themselves useful in the hands of skilled men. The incentive for Dr. Marshall writing this paper was certain unqualified statements made at the last meeting of the A. V. M. A. in the discussions. One was that "90 per cent. of the drugs we use in our practice are fakes." Another one was "that the firing-iron should never be used." Dr. Marshall would like to know whether the veterinary profession is ready to accept these statements, and, furthermore, if we accept them as factors, then, in his opinion, it is about time to revise the pharmacopœia.

The next paper, which was very educating to all, was the "Anatomo-pathological Study of Ringbone and Spavin as Indicated by Examination of Pathological Specimens," by Dr. S. J. J. Harger. The subject was one that the author was in thorough touch with, and he covered the ground in a very interesting and instructive manner.

Reports of progress from several of the committees were received.

E. M. Ranck,
Secretary.

---

### AMERICAN VETERINARY MEDICAL ASSOCIATION.

President Winchester has appointed the following members of the Committee on Pharmacopœia: Dr. L. A. Merillat, Chicago, chairman; Dr. E. L. Quitman, Chicago; Dr. D. King Smith, Toronto; Dr. J. J. Repp, Iowa; Dr. E. M. Ranck, Philadelphia; Dr. H. D. Hanson, New York; Dr. R. R. Bell, of Brooklyn.

President Winchester has appointed Dr. S. L. Hunter, of Fort Leavenworth, Kansas, to fill the vacancy on the Army Legislative Committee caused by the death of Dr. R. S. Huidekoper.

